# Intraneural Synovial Cyst of the Common Peroneal Nerve: An Unusual Cause of Foot Drop with Four-Year Follow-Up

**DOI:** 10.1155/2019/8045252

**Published:** 2019-08-05

**Authors:** Abrar Adil, Clint Basener, Jake Checketts

**Affiliations:** ^1^Department of Orthopaedic Surgery, Oklahoma State University Medical Center, USA; ^2^Center for Health Sciences, Oklahoma State University, USA

## Abstract

In our case report, we describe a 55-year-old male patient with isolated foot drop due to an intraneural synovial ganglion. We successfully treated the lesion with decompression via epineurotomy combined with primary division of the recurrent articular branch of the common peroneal nerve (CPN). Compression neuropathies of the common peroneal nerve arise from a variety of causes. Intrinsic compression due to intraneural ganglion cysts of the CPN is rare. Previous reports of simple decompression of the cystic fluid have resulted in recurrence. The unified articular theory describes a pathway for fluid to fill from the proximal tibiofibular joint into the CPN via a recurrent articular branch. In our case, we divide this articular branch which we feel prevents recurrence.

## 1. Introduction

Peripheral entrapment neuropathies are defined as a state of altered transmission in a peripheral nerve due to physical irritation from adjacent anatomic structures. The course of the common peroneal nerve (CPN) as it courses around the proximal end of the fibula lends it to entrapment and mechanical constriction. This constriction may be due to direct trauma, as well as external or internal pressures, or indirect factors that alter the normal biomechanics of the knee.

In this report, we describe a rare cause of peroneal nerve entrapment due to increased pressure within the nerve sheath due to an intraneural ganglion. Currently, it is believed that such intraneural ganglia occur by a retrograde flow of synovial fluid within the epineurium from a small branch of the CPN, the recurrent articular branch [1–6]. This branch arises millimeters distal to the bifurcation of the CPN and travels to the superior tibiofibular joint. This connection results in a direct conduit for synovial fluid to flow into the nerve.

Literature regarding intraneural ganglia is limited and exists primarily in the neurosurgical literature. A previous case report where intraneural ganglia of the CPN were treated with decompression of the nerve via epineurotomy had delayed recurrence [7]. This finding led to ligation of the recurrent articular branch with resolution of symptoms. Other reports of foot drop due to compression of the CPN by an intraneural ganglia indicate full recovery of CPN function is possible if diagnosed early, identified precisely, and promptly treated [8–10]. We chose to primarily divide the recurrent articular branch after manual decompression of the CPN.

## 2. Case Presentation

**Figure 1:**
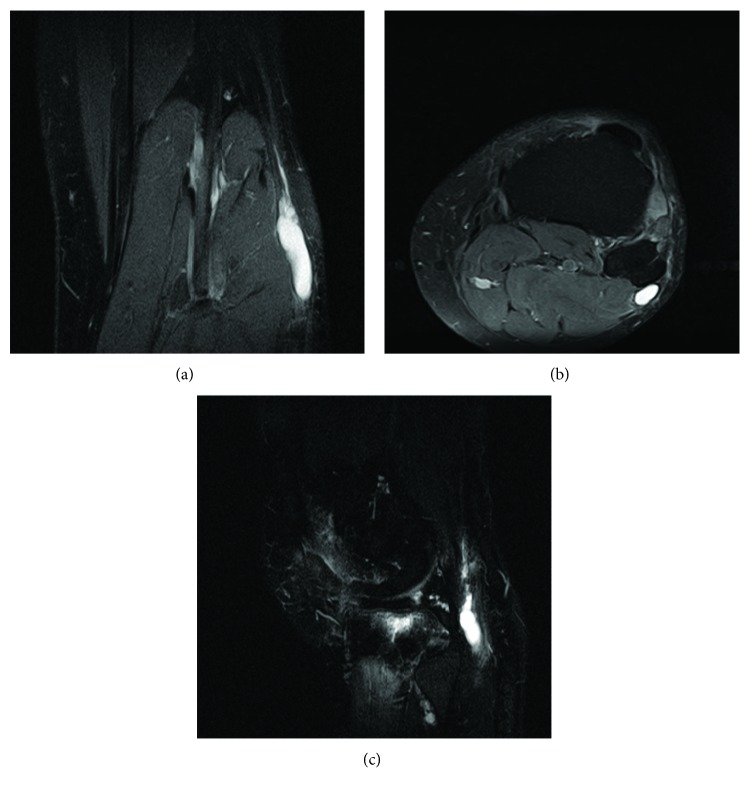
3 MRI views (sagittal, coronal, and axial) of the knee revealing a hyperintense fluid collection within the CPN with connection to the proximal tibiofibular joint, consistent with an intraneural ganglion cyst.

A 55-year-old male college professor presented to us with an 8-month history of progressive gait abnormality due to ankle weakness with eventual foot drop. He denies any recent or remote history of trauma to the lower extremity. He experienced no pain and minimal paresthesias. Examination in the office revealed ankle dorsiflexion strength of 1/5 and similar weakness with isolated eversion of the foot. EMG was obtained with delayed conduction across the CPN. MRI of the knee revealed a hyperintense fluid collection within the CPN with connection to the proximal tibiofibular joint, consistent with an intraneural ganglion cyst (Figure 1).

He had tried numerous conservative means and had been referred to several specialties prior to presenting to our office. Due to the progressive neurological deficit, we opted to proceed with surgical treatment without delay. The patient was informed that data from the case may be collected and submitted for publication to which he gave consent.

**Figure 2:**
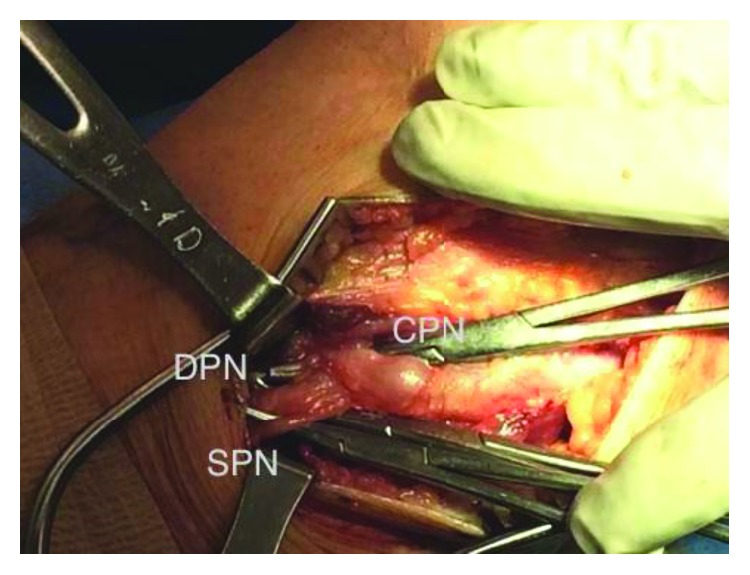
The distended common peroneal nerve is seen at its bifurcation. CPN: common peroneal nerve; DPN: deep peroneal nerve; SPN: superficial peroneal nerve.

The patient was placed in a supine position with a bump under the ipsilateral PSIS. A linear longitudinal incision was utilized along the posterior border of the fibular head distally and extended proximally along the posterior border of the biceps femoris tendon. The CPN was identified deep and posterior to the biceps femoris tendon and was mobilized by releasing adherent fibers of the peroneus longus and its fascia. Neurolysis was performed distally and proximally. The distended CPN was traced to its bifurcation, and the deep and superficial branches were visualized (Figure 2). The distended recurrent articular branch was found to arise just proximal to the CPN and then coursed anteriorly toward the proximal tibiofibular joint.

**Figure 3:**
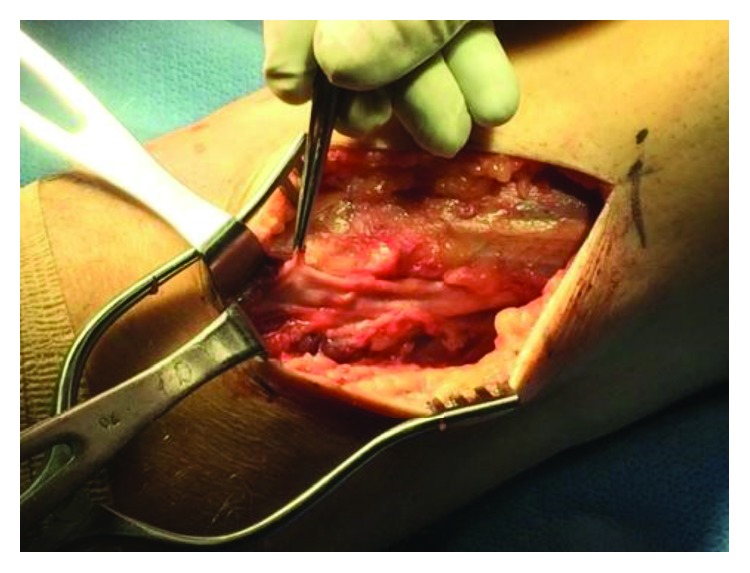
The recurrent articular branch stump after ligation and resection.

We opted to perform an epineurotomy with gentle manual decompression of the distended CPN. Approximately 15-20 mL of clear, gelatinous fluid was expressed from the epineurotomy. To minimize chance of recurrence, primary ligation and resection of the recurrent articular branch of the CPN was performed (Figure 3).

**Figure 4:**
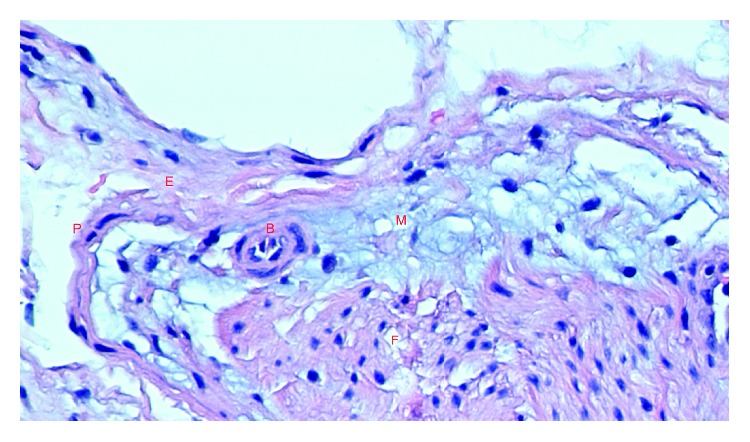
Hematoxylin and eosin-stained high-power photomicrograph. Cross-section of resected intraneural ganglion cyst. P: perineurium; E: epineurium; B: blood vessel; M: mucin. F: fascicle. Stringy grey-blue-stained mucin is seen within the epineural layer consistent with an intraneural ganglion cyst.

After ligation of the recurrent articular branch (Figure 3), it was sent for histopathological examination along with the expressed fluid. The report confirmed an intraneural ganglion cyst with normal-appearing nerve fascicles and no evidence of metaplasia (Figure 4).

The wound was closed over a drain, which was discontinued by the patient at 24 hours. A soft dressing was applied without splint to allow early range of motion to tolerance.

Postoperatively, interval improvement in motor strength recovery was noted to be slow. Pain and paresthesia, which are often seen in other similar cases, were absent in this case and may have made clinical recovery less dramatic. At 3-month follow-up, he had regained 4-4+/5 ankle dorsiflexion and 3+/5 eversion without clinical evidence of recurrence.

After four years of follow-up, the patient is currently very satisfied with the results of his treatment. He has normal function and 5/5 strength bilaterally. Furthermore, we asked the patient a series of questions consistent with the Western Ontario McMaster Universities Osteoarthritis Index (WOMAC) [11] to assess his pain, stiffness, and physical function postoperatively. We asked the patient to rate his pain, stiffness, and physical function 0-4 for all questions within the WOMAC scale with 0 being no pain, stiffness, or disfunction and 4 being extreme pain, stiffness, or disfunction. The patient reported a “0” for each question, indicating he had no pain, stiffness, or disfunction. The patient's only current complaint at the 4-year follow-up is a small area (2 × 2 cm) of paresthesia on the dorsal surface of the foot that does not affect him negatively in any way.

## 3. Discussion

The common peroneal nerve arises in the popliteal fossa from the sciatic nerve. It then passes through the origin of the peroneus longus muscle and divides into deep and superficial branches. The superficial branch innervates the peroneal muscles and supplies the skin of the anterolateral leg and dorsal foot. The deep branch runs in the anterior compartment and innervates the dorsiflexors of the toes and foot with a terminal cutaneous branch supplying the first web space. The nerve is vulnerable to compression in its course over the fibular neck. It is the most commonly entrapped nerve in the leg. In this case, it is important to realize that an intrinsic rather than an extrinsic pressure results in the neuropraxia and symptoms.

The unified articular theory as defined by Spinner et al. explains the method by which an articular branch of the CPN serves as a synovial conduit for fluid to enter the nerve [2]. This theory suggests that the intraneural ganglion cysts form around synovial joints and then spread to nearby nerves via a capsular rent into an articular branch of the nerve (in our case, the CPN). Distension and fluid pressure within the nerve sheath may cause neuropathic pain, paresthesias, and/or paresis.

As seen in our case, intraneural ganglion cysts can be readily identified via MRI, and this type of imaging can also be useful in the morphologic classification of intraneural ganglion cysts [12]. Furthermore, authors have described three identifiers on MRI that are highly suggestive of intraneural ganglion cysts, which are useful for differentiating these cysts from their extraneural counterparts. The first of which is the “transverse limb sign,” which assesses for a peroneal intraneural cyst with extension along the transverse limb of the articular branch of the peroneal nerve, resulting in a horizontal, linear area of increased T2 signal along the course of the nerve branch [3]. This sign was found by Spinner et al. to be 100% sensitive and specific for intraneural ganglion cysts of the peroneal nerve. The next is the “signet ring sign,” which is described as the eccentric displacement of fascicles by a cyst within the epineurium [3]. This sign was found by Spinner et al. to be 100% sensitive and 86% specific for intraneural ganglion cysts of the peroneal nerve. Lastly, the “tail sign” assesses for a joint connection between the nerves. This connection could represent either the articular branch (in intraneural ganglia) or nonneural pedicles (in extraneural ganglia) [3]. This sign was 100% sensitive for identifying joint connections but could not distinguish between intra- and extraneural cysts.

The rarity of this lesion often leads to delay in diagnosis, as time to definitive referral may be significant, and as such, the role of conservative treatment in this case was minimal. It should also be noted that intraneural ganglion cysts can occur in the pediatric population. Although our patient was not a pediatric patient, surgeons and clinicians should be aware of this possibility in children to ensure early detection. Motor recovery after surgery is typically more favorable in the pediatric population with early treatment as compared to adults [13, 14].

## 4. Summary

We describe an uncommon cause of foot drop without other symptoms of nerve compression such as pain or paresthesia. MRI and EMG were employed to aid in isolation of the cause of CPN compression. Decompression of the intraneural ganglion cyst with division of the recurrent articular branch provides a method of preventing recurrence of the nerve compression.

## 5. Conclusion

Paresis of muscles about the ankle carries a wide differential diagnosis including spinal pathologies and peripheral nerve compression. The presence of foot drop and a clinical picture consistent with CPN compression are confirmed with electromyography. The addition of MRI differentiates between an entrapment neuropathy and cystic intraneural ganglia arising from the proximal tibiofibular joint.

Once the diagnosis of an intraneural ganglion cyst is made in the presence of neurological deficit, successful surgical treatment should include division of the recurrent articular branch to minimize the chances of recurrence. Decompression alone may lead to recurrence, and revision surgery is often met with adhesions and may place the CPN and its branches at risk. Our case demonstrates that even those with significant symptoms may make a full recovery following operative management.
